# Motivation, self-evaluation, performance: comparison of objective and subjective health indicators in higher education

**DOI:** 10.3389/fspor.2026.1831329

**Published:** 2026-05-08

**Authors:** Balázs Fügedi, Anita Molnár, Erika Beregi

**Affiliations:** 1 Institute of Sport Science, Eszterházy Károly Catholic University, Eger, Hungary; 2 Sport and Health Research Group, Eszterházy Károly Catholic University, Eger, Hungary; 3 Institute of Physical Education and Sport Science, University of Nyíregyháza, Nyíregyháza, Hungary; 4 Faculty of Health Science, University of Miskolc, Miskolc, Hungary

**Keywords:** health behavior, health promotion, higher education, life transitions, physical activity, sports motivation, well-being, young adulthood

## Abstract

Health-conscious behavior, culture, and motivation positively impact physical activity. However, compared to high school, university students’ daily physical exercise shows a declining trend, with non-sporting students generally less satisfied with their physical condition. This study compares the sports motivation, subjective health, and fitness status of sports major and non-sports major university students, contrasting self-evaluation data with objective measurements. Following purposive sampling, 146 individuals participated (sports major students: *n* = 50, non-sports major students: *n* = 96); the uneven group distribution constitutes a limitation of the study. The average age was 21.33 ± 2.19 years, comprising 35.9% females and 64.1% males. Fitness self-assessment utilized a self-report questionnaire (32 items; Cronbach's alpha = 0.658), alongside the objective determination of body mass index (BMI) using bioelectrical impedance analysis (InBody 720, Biospace Co., Seoul, South Korea), estimated maximal oxygen uptake (estimated VO2max) via the 12-minute Cooper run test, and functional movement quality assessed with the Functional Movement Screen (FMS). Resultsindicated that 35.5% of non-sports majors and 81.3% of sports majors judged their performance capacity and fitness as good or very good. Sports majors demonstrated significantly higher estimated VO2max values (39.39 ± 6.81 vs. 28.23 ± 9.79 mL/kg/min; *p* < 0.001), while BMI values were nearly identical across groups (24.03 ± 4.90 vs. 24.01 ± 3.34 kg/m^2^; *p* = 0.980). Self-evaluations regarding performance and fitness aligned with objective measurements, confirming the superior cardiovascular condition of sports majors. BMI values were similar across groups; however, this should not be interpreted as direct evidence of health-conscious behavior, as weight maintenance may reflect a range of factors unrelated to intentional health behavior. These findings contribute to the understanding of physical activity determinants during the transition into higher education.

## 1 Introduction

Research-supported correlations can be observed between lifestyle-related risk factors, such as inactivity, unhealthy diet, smoking, alcohol consumption, and the development of chronic diseases (1). Establishing a health-conscious habit system plays a prominent role in the lives of children and young people. It is important that the practice of positive health behavior and regular physical exercise become self-evident for them (2). At the same time, the transition from adolescence into higher education (early adulthood) represents a critical life transition where physical activity often sharply declines. During this vulnerable period, students lead an increasingly inactive lifestyle, thereby creating the possibility for the development of numerous health problems (3, 4). Understanding how social and behavioral influences interact to hinder or support engagement during this phase is essential for designing policies and programs that promote sustainable, active lifestyles.

The effectiveness of physical exercise has been proven in the prevention and treatment of psychological and somatic diseases, and it also offers numerous opportunities in health promotion (5). However, it should be highlighted that there are many influencing factors behind regularly performed, committed sports activities. These include, for example, the athlete's age, gender, relationship with their family, and the parents’ sports background. Even more complex processes lie behind the commitment to sports, where the individual's characteristics and strengths also play a role (6). In the case of regularly sporting adolescents, unhealthy habits are less frequently observed, unlike their non-sporting peers. Based on all this, it can be stated that organized sports are a prominent arena and opportunity for shaping health behavior (7). In this context, higher education institutions represent a similarly critical arena, as they simultaneously concentrate a large population of young adults at risk of physical activity decline and possess the structural capacity to implement health-promoting programs and supportive environments.

The role of sports motivation should be highlighted, as it functions as a driving force. All this is also necessary regarding the extent and quality of commitment to sports (8). During the examination of sports motivation, it is essential to distinguish between “intrinsic” and “extrinsic” motivation. In the former, the enjoyment derived from the action appears as motivation. The main goal of the latter is prevention and increasing fitness. Those who do not engage in sports activities often indicate a lack of motivation as the reason for inactivity. At the same time, one's own internal determination mainly plays a role in the background of starting sports (9). Szerdahelyi’s (10) research pointed out that those students who judge that they perform the amount of physical activity recommended by the World Health Organization also feel their health status is better. In young people's decisions supporting sports, the opportunities for establishing social relationships, external appearance, the improvement of their psychological state, as well as the preservation and development of their health play an essential role.

Previous research highlighted the differences between the subjective well-being of adolescent sporting and non-sporting young people. It was found that doing sports had a positive effect on young people's opinions about their health and their psychological well-being. Movement appeared as a source of joy for them, and its positive mental and social effects also prevailed. Besides this, it also provided significant support in the successful management of stressful situations (11). The results of the research conducted by Lukács and co-worker (12) among sports major and non-sports major university students showed that only the physical activity number of sports majors complies with the recommendation of the World Health Organization. The physical activity level of non-sports majors is significantly lower than what would be appropriate for their age and the recommendations.

Furthermore, family socialization shapes long-term behavioral habits. Numerous studies conducted among the 10–14 age group have pointed out that parents’ attitude towards sports plays a decisive role in children's physical activity. It has been proven that if parents perform regular sports activities, it also increases the child's activity. If the parents are inactive, it also increases the probability of the children's inactivity. It should be highlighted that similar results can be observed regarding both parental modeling (13, 14). Shared sports activities with the family provide an excellent opportunity for value transmission. It is important that the father's sporting pattern and supportive grandparental activity also have an impact on the child's sporting habits (15). At the same time, Fenyves et al. (16), examining sporting habits and sports motivation, found that in the case of Hungarian university students, the family played less of a role as a motivating factor promoting sports. However, Kinczel et al. (9) emphasized the importance of the parents’ role and exemplary behavior in their study. The children of sporting parents are more likely to become athletes compared to the children of non-sporting parents.

Despite the growing body of research on physical activity during the transition to higher education, few studies combine subjective self-evaluation with multiple objective physical measurements (BMI/BIA, estimated VO2max, and FMS) to examine both the magnitude and quality of physical fitness differences between sports major and non-sports major students. Unlike studies that rely on a single measurement tool, this study integrates questionnaire data with three objective indicators — BMI/BIA, estimated VO2max, and FMS — allowing a more comprehensive comparison of physical status and health consciousness between student groups.

The aim of the research is to compare the sports motivation, subjective health, and fitness status of sports major and non-sports major university students and to contrast self-evaluation data with objective measurement results. Based on the reviewed literature, the following hypotheses were formulated:

H1: Sports major students will demonstrate significantly higher estimated VO2max values than non-sports major students.
H2: Sports major students will report more favorable subjective self-evaluations across fitness, health, and well-being dimensions compared to non-sports major students.
H3: Subjective self-evaluations will correlate significantly with objective physical indicators (estimated VO2max and BMI), but not with functional movement quality (FMS scores).
H4: Parental sports background will not directly determine group membership (sports major vs. non-sports major), though indirect associations with social influences on sports motivation may still be present.

## 2 Materials and methods

### 2.1 Participants and recruitment

The study employed a purposive sampling strategy, a non-probability sampling method in which participants are selected based on predefined criteria relevant to the research objectives (17). Data were collected during the autumn semester of the 2025/2026 academic year (October–November 2025). Following this procedure, 146 individuals participated in the study (sports major students: *n* = 50, non-sports major students: *n* = 96). The average age of the participants was 21.33 ± 2.19 years, 35.9% were female, and 64.1% were male. Participants were recruited from a rural Hungarian higher education institution enrolling over 3,000 full-time students across a wide range of disciplines, including social sciences, natural sciences, and teacher training. The student body draws from a wide geographic catchment area, with students originating from diverse regions across Hungary. Sports major students were defined as those enrolled in a Sport Science Bachelor of Science (BSc) programme, actively participating in compulsory practical courses, and holding official competition accreditation issued by a recognized sports federation. Non-sports major students were defined as those not enrolled in any sport science programme, without competition accreditation or formal sports affiliation, but self-reporting engagement in some form of recreational physical activity. This inclusion criterion was applied deliberately, as the study aimed to examine subjective self-assessment of physical status; participants without any physical activity experience would lack a meaningful reference frame for the self-evaluation items. The research was conducted under the ethical regulations of the local institutional review board and conformed to the directives of the Helsinki Declaration. Written consent was obtained from all participating students prior to the study, and it was explicitly emphasized that participants could withdraw from the research at any time if they chose to do so. As a purposive sampling strategy was employed, all eligible students who met the predefined inclusion criteria and were invited to participate completed the study.

### 2.2 Questionnaire

Data on subjective health status, physical fitness perception, sports motivation, parental sports history, and addictive habits were collected using a self-report, paper-based questionnaire completed by hand. The instrument comprised 32 items covering demographic characteristics, self-perceived physiological and fitness status, subjective health evaluation, sports motivation, parental sports background, and lifestyle and addictive behaviors. Responses were recorded on 5-point and 7-point Likert scales, supplemented by ranking tasks and open-ended questions where appropriate. The questionnaire was not validated as a complete instrument prior to this study; however, individual items were selected and adapted from previously validated questionnaires used in health behavior and sports motivation research (e.g., 6, 8–10). The internal consistency reliability of the questionnaire was assessed using Cronbach's alpha, which yielded a value of 0.658. This value falls at the lower boundary of acceptable reliability (18), and is acknowledged as a methodological limitation of the present study.

### 2.3 Objective measurements

Body composition was assessed using the InBody 720 multi-frequency bioelectrical impedance analyser (Biospace Co., Ltd., Seoul, South Korea), which employs an 8-point tactile electrode system and measures at six different frequencies (1, 5, 50, 250, 500, and 1,000 kHz). The device provides segmental body composition data including body mass, body fat percentage, fat-free mass, and body mass index (BMI). Measurements were conducted under standardised conditions, with participants abstaining from food and vigorous exercise for at least two hours prior to testing. Estimated maximal oxygen uptake (estimated VO2max) was determined using the 12-minute Cooper run test (19), conducted on a standard 400-metre athletic track under standardised conditions. Participants were instructed to cover the greatest possible distance within 12 min. VO2max was estimated using the original Cooper equation:

VO2max(mL/kg/min)=(distanceinmetres-504.9)/44.73

This formula was developed and validated by Cooper (19) and has been widely used in population-based fitness assessments. Test administration was supervised by qualified sports science professionals. Functional movement quality was assessed using the Functional Movement Screen (FMS; 20), a standardised battery of seven fundamental movement pattern tests: deep squat, hurdle step, inline lunge, shoulder mobility, active straight-leg raise, trunk stability push-up, and rotary stability. Each movement was scored on a 0–3 ordinal scale, yielding a composite FMS score ranging from 0 to 21. Testing was conducted using the official FMS equipment and followed the standardised protocol described by Cook et al. (20). FMS assessments were administered by a trained sports science professional who is one of the authors of the present study, ensuring consistent application of the standardised protocol throughout the entire data collection period.

### 2.4 Statistical analysis

The data were processed using IBM SPSS Statistics, Version 21.0 (IBM Corp., Armonk, NY, USA). During descriptive statistics, the mean and standard deviation of the examined variables were calculated for both groups (sports major and non-sports major). To examine the differences between the two independent groups, an Independent Samples t-test was applied for all quantitative variables. Before applying the t-test, the homogeneity of variances was checked with Levene's test. When evaluating significance, depending on the result of Levene's test, the rows assuming equal or unequal variances were taken into account. The threshold for statistical significance was set at *p* < 0.05. For the comparison of categorical variables, Pearson's Chi-square test was applied to explore associations. To examine how closely subjective self-evaluation relates to objective physical indicators, Pearson correlation analysis (Pearson's r) was performed.

## 3 Results

The analysis of the collected data aimed to explore differences between sports major and non-sports major student groups, to examine the potential influencing role of family background, and to understand the correlations between objective and subjective status indicators. For all t-test comparisons, Cohen's d was calculated as a measure of effect size, and 95% confidence intervals (CI) are reported for the mean differences.

### 3.1 Group differences along objective and subjective indicators

The comparison of the two independent groups (sports major vs. non-sports major) was performed with an independent samples t-test (Table 1). For each comparison, Cohen's d was calculated as a measure of effect size, and 95% confidence intervals (CI) are reported for the mean differences to provide a more complete statistical picture.

**Table 1:** Comparison of physical and subjective indicators of sports major and non-sports major students.

Variable	Non-sports major (*N* = 96) M ± SD	Sports major (*N* = 50) M ± SD	t-value	*p*-value	d	Effect size	CI Lower	CI Upper
Physical indicators								
BMI (kg/m^2^)	24.03 ± 4.90	24.01 ± 3.34	0.025	0.980	−0.00	Negligible	−1.55	1.51
Estimated VO2max (mL/kg/min)	28.23 ± 9.79	39.39 ± 6.81	−8.004	*** < 0.001	1.26	Large	8.10	14.22
FMS	18.24 ± 2.12	17.28 ± 4.20	1.527	0.132	−0.32	Small	−1.99	0.07
Self-evaluation								
Fitness	3.79 ± 1.08	4.24 ± 0.60	−3.263	**0.001	0.48	Small	0.12	0.78
Endurance	3.78 ± 1.06	4.04 ± 0.35	−2.179	*0.031	0.29	Small	−0.05	0.57
Own performance	3.23 ± 0.85	4.13 ± 0.70	−6.702	*** < 0.001	1.12	Large	0.62	1.18
Performance compared to peers	3.11 ± 0.94	3.88 ± 0.63	−5.789	*** < 0.001	0.91	Large	0.48	1.06
Own endurance	3.21 ± 0.91	3.63 ± 0.73	−3.052	**0.003	0.49	Small	0.13	0.71
Endurance compared to peers	3.27 ± 0.97	3.78 ± 0.62	−3.801	*** < 0.001	0.59	Medium	0.21	0.81
Own health status	3.55 ± 0.99	4.20 ± 0.58	−4.991	*** < 0.001	0.75	Medium	0.35	0.95
Health compared to peers	3.56 ± 1.00	4.10 ± 0.59	−4.079	*** < 0.001	0.61	Medium	0.24	0.84
General well-being	3.65 ± 0.91	4.08 ± 0.79	−2.996	**0.003	0.49	Small	0.13	0.73
Filled with energy	3.26 ± 1.00	3.86 ± 0.65	−4.345	*** < 0.001	0.67	Medium	0.29	0.91
Social/Information sources								
Friends	3.17 ± 1.23	3.80 ± 0.96	−3.392	**0.001	0.55	Medium	0.24	1.02
Entertainment	3.25 ± 1.15	4.02 ± 0.92	−4.359	*** < 0.001	0.71	Medium	0.40	1.14
Parent	2.42 ± 1.33	3.27 ± 1.24	−3.724	*** < 0.001	0.65	Medium	0.40	1.30
Professional	1.86 ± 1.11	3.12 ± 1.18	−6.178	*** < 0.001	1.11	Large	0.87	1.65
Family background								
Father played sports previously	1.43 ± 0.50	1.48 ± 0.51	−0.607	0.545	0.10	Negligible	−0.12	0.22
Mother played sports previously	1.57 ± 0.61	1.72 ± 0.45	−1.644	0.103	0.27	Small	−0.04	0.34

M, mean; SD, standard deviation; d, Cohen's d (effect size); CI Lower and CI Upper, lower and upper bounds of the 95% confidence interval of the mean difference (Sports major−Non-sports major). Positive CI values indicate higher scores in sports major students; negative values indicate higher scores in non-sports major students. Independent samples t-test was applied for all comparisons. Effect size benchmarks follow Cohen (24): negligible (d < 0.20), small (d ≥ 0.20), medium (d ≥ 0.50), large (d ≥ 0.80). Significance levels: * *p* < 0.05; ** *p* < 0.01; *** *p* < 0.001.

Regarding objective physical measurements, a mixed picture emerged. A statistically highly significant difference was detected in the estimated VO2max value, the primary indicator of cardiovascular endurance. The mean value of sports major students (M = 39.39 ± 6.81 mL/kg/min) significantly exceeded that of non-sports major students [M = 28.23 ± 9.79 mL/kg/min; t(144) = −8.004, *p* < 0.001, d = 1.26, 95% CI [8.10, 14.22] mL/kg/min], representing a large effect. In contrast, body mass index values were practically identical between the two groups [M = 24.01 ± 3.34 vs. M = 24.03 ± 4.90 kg/m^2^; t(144) = 0.025, *p* = 0.980, d = −0.00, 95% CI [−1.55, 1.51] kg/m^2^], indicating a negligible effect with no practical or statistical significance. Similarly, no significant difference was found between the FMS scores measuring functional movement quality [M = 17.28 ± 4.20 vs. M = 18.24 ± 2.12; t(144) = 1.527, *p* = 0.132, d = −0.32, 95% CI [−1.99, 0.07]], representing a small, non-significant effect.

In contrast to the objective measurements, sports major students reported significantly more favorable values across all examined dimensions of subjective self-evaluation. The largest difference was observed in the assessment of own performance, where sports majors rated themselves considerably higher [M = 4.13 ± 0.70 vs. M = 3.23 ± 0.85; t(144) = −6.702, *p* < 0.001, d = 1.12, 95% CI [0.62, 1.18]], a large effect. Large effects were also observed for performance compared to peers [M = 3.88 ± 0.63 vs. M = 3.11 ± 0.94; t(144) = −5.789, *p* < 0.001, d = 0.91, 95% CI [0.48, 1.06]]. Medium effects were recorded for own health status [M = 4.20 ± 0.58 vs. M = 3.55 ± 0.99; t(144) = −4.991, *p* < 0.001, d = 0.75, 95% CI [0.35, 0.95]], filled with energy [t(144) = −4.345, *p* < 0.001, d = 0.67, 95% CI [0.29, 0.91]], endurance compared to peers [t(144) = −3.801, *p* < 0.001, d = 0.59, 95% CI [0.21, 0.81]], health compared to peers [t(144) = −4.079, *p* < 0.001, d = 0.61, 95% CI [0.24, 0.84]]. Small effects, though still statistically significant, were found for fitness [t(144) = −3.263, *p* = 0.001, d = 0.48, 95% CI [0.12, 0.78]], own endurance [t(144) = −3.052, *p* = 0.003, d = 0.49, 95% CI [0.13, 0.71]], and endurance [t(144) = −2.179, *p* = 0.031, d = 0.29, 95% CI [−0.05, 0.57]] and general well-being [t(144) = −2.996, *p* = 0.003, d = 0.49, 95% CI [0.13, 0.73]]. It is noted that the CI for the Endurance variable marginally crosses zero, indicating that this result should be interpreted with caution despite reaching statistical significance.

A similar trend emerged regarding the perceived importance of social and information sources. Sports major students attributed significantly greater importance to professional opinion [t(144) = −6.178, *p* < 0.001, d = 1.11, 95% CI [0.87, 1.65]], a large effect, as well as to entertainment [t(144) = −4.359, *p* < 0.001, d = 0.71, 95% CI [0.40, 1.14]], parents [t(144) = −3.724, *p* < 0.001, d = 0.65, 95% CI [0.40, 1.30]], and friends [t(144) = −3.392, *p* = 0.001, d = 0.55, 95% CI [0.24, 1.02]], all representing medium effects. In all cases, differences were in favor of the sports major group.

To evaluate whether the observed group differences could be attributed to potential confounders, additional analyses of covariance (ANCOVA) were conducted with age, sex, and BMI entered as covariates. The group difference in estimated VO2max remained highly significant after adjustment [F(1,140) = 34.095, *p* < 0.001, partial *η*^2^ = 0.196], with adjusted mean values of 28.84 mL/kg/min for non-sports majors and 38.19 mL/kg/min for sports majors (95% CI for the mean difference: 6.19 to 12.52 mL/kg/min). Similarly, the advantage of sports majors persisted for key subjective outcomes: own performance [F(1,139) = 39.833, *p* < 0.001, partial *η*^2^ = 0.223], performance compared to peers [F(1,140) = 20.954, *p* < 0.001, partial *η*^2^ = 0.130], own health status [F(1,140) = 19.994, *p* < 0.001, partial *η*^2^ = 0.125], health compared to peers [F(1,140) = 13.719, *p* < 0.001, partial *η*^2^ = 0.089], own endurance [F(1,140) = 10.020, *p* = 0.002, partial *η*^2^ = 0.067], endurance compared to peers [F(1,140) = 10.695, *p* = 0.001, partial *η*^2^ = 0.071], and general well-being [F(1,140) = 8.702, *p* = 0.004, partial *η*^2^ = 0.059].

### 3.2 Examination of the role of family background

Pearson's chi-square analyses were conducted to examine whether parental sports background was associated with group membership and selected categorical variables (Table 2). Neither father's sports background ($\chi^{2}$ = 0.373, df = 1, *p* = 0.542) nor mother's sports background ($\chi^{2}$ = 5.118, df = 2, *p* = 0.077) showed a significant association with whether the student belonged to the sports major or non-sports major group. In addition, significant associations were identified between father's sports background and the importance attributed to parental opinion ($\chi^{2}$ = 10.090, df = 4, *p* = 0.039) and professional opinion ($\chi^{2}$ = 10.976, df = 4, *p* = 0.027). A further significant association was found between mother's sports background and the evaluation of endurance ($\chi^{2}$ = 18.115, df = 8, *p* = 0.020).

**Table 2:** Examination of relationships between categorical variables (Pearson's Chi-square test).

Examined variables	*χ* ^2^	df	*p*-value	Cramér's V	Result
Main research questions					
Group membership × Father played sports previously	0.373	1	0.542	**0**.**051**	Not significant
Group membership × Mother played sports previously	5.118	2	0.077	**0**.**187**	Not significant
Further revealed correlations					
Father's sports background × Parental opinion	10.090	4	0.039	**0**.**264**	Significant
Father's sports background × Professional opinion	10.976	4	0.027	**0**.**275**	Significant
Mother's sports background × Evaluation of endurance	18.115	8	0.020*	**0**.**250**	Significant*

χ^2^ (Chi-square): The value of the test statistic.

df (Degrees of freedom): Degrees of freedom.

*p*-value (Sig.): Significance level. Values in bold indicate a statistically significant relationship (*p* < 0.05).

* The conditions of the test were violated (too many cells had an expected frequency below 5), so this result should be treated with caution.

Taken together, these findings suggest that parental sports background did not directly distinguish sports major from non-sports major students in this sample, but it was related to selected motivational and self-evaluative dimensions.

### 3.3 Correlation relationships between objective and subjective indicators

To examine the consistency between objective physical condition and subjective self-perception, Pearson correlation analysis was applied. The results indicated that students’ self-evaluations are partially grounded in objective indicators. The estimated VO2max showed a significant, moderately strong positive correlation with most subjective variables, most strongly with the evaluation of own performance (r = 0.345, *p* < 0.001) and performance compared to peers (r = 0.344, *p* < 0.001). BMI showed a significant, weak-to-moderate negative relationship with several self-evaluation indicators, most notably endurance compared to peers (r = −0.261, *p* = 0.002) and own health status (r = −0.211, *p* = 0.011).

The FMS score, measuring functional movement quality, did not show a significant correlation with any subjective variable. This indicates that movement quality, as opposed to cardiovascular endurance or body composition, does not appear to form the basis of students’ subjective fitness or health perception.

The subjective self-evaluation variables demonstrated high internal consistency, with strong and significant positive intercorrelations (e.g., Fitness and Endurance: r = 0.638, *p* < 0.001), suggesting the presence of a general positive self-image factor among the participants.

## 4 Discussion

The aim of the present research was to compare the physical status, subjective self-evaluation, and sports motivation of sports major and non-sports major university students, with particular attention to the extent to which subjective judgments reflect objective performance indicators.

H1 was confirmed. The significantly higher estimated VO2max values of sports major students (d = 1.26, a large effect) are consistent with the expected consequences of structured, regular physical activity embedded in their study programme. This result aligns with the findings of Lukács and Suszter (12), who reported that only sports major students at the same institution met the physical activity recommendations of the World Health Organization, while the activity levels of non-sports majors remained substantially below age-appropriate guidelines. It is plausible that the regular, professionally supervised physical training inherent to a Sport Science BSc programme contributes substantially to superior cardiovascular fitness; however, the cross-sectional design of this study does not permit causal inference in this regard.

H2 was also confirmed. Sports major students reported more favorable subjective self-evaluations across all examined dimensions, with effect sizes ranging from small (Endurance, d = 0.29) to large (Own performance, d = 1.12; Performance compared to peers, d = 0.91). This pattern is consistent with prior research by Pfau and Mészáros (21), according to which non-sporting university students demonstrate lower satisfaction with their physical condition. In the present study, only 35.5% of non-sports major students evaluated their own fitness positively, compared to 81.3% of sports majors. While this difference is substantial, alternative explanations cannot be excluded — for instance, sports major students may report higher self-evaluations partly due to the social norms and identity associated with their academic environment, rather than solely due to their objective physical superiority. This possibility warrants consideration in future research employing longitudinal or experimental designs.

Importantly, the group differences in VO2max and in the main subjective self-evaluation indicators remained statistically significant after adjustment for age, sex, and BMI in ANCOVA models, with small to large partial *η*^2^ values. This suggests that the observed advantages of sports major students in both objective and subjective domains cannot be attributed solely to basic demographic or anthropometric differences.

H3 was partially confirmed (partially, in that correlations were moderate rather than strong). The estimated VO2max showed moderate positive correlations with most subjective self-evaluation variables (strongest for own performance: r = 0.345, *p* < 0.001), and BMI showed weak-to-moderate negative associations with several indicators (strongest for endurance compared to peers: r = −0.261, *p* = 0.002). These findings suggest that students’ self-perceptions are partially grounded in real performance differences, which is consistent with Szakály et al. (22) and Bartha and Bácsné Bába (23). However, the magnitude of these correlations is moderate rather than strong, indicating that factors beyond objective physical performance — such as social comparison, motivational orientation, or psychological well-being — also shape subjective self-evaluation.

A particularly notable finding concerns functional movement quality, measured by the FMS. The FMS score did not significantly differ between groups (d = −0.32, a small non-significant effect) and showed no significant correlation with any subjective variable. This suggests that movement quality, unlike cardiovascular endurance or body composition, is not incorporated into students’ subjective health or fitness self-image. Young people appear to evaluate their physical status primarily through performance-based dimensions (e.g., running capacity) and external appearance (e.g., body weight), while functional movement competency remains largely outside their subjective awareness. This is an observational finding; no causal conclusion can be drawn about whether improving FMS awareness would change subjective health perception.

Regarding sports motivation, health preservation and fitness development emerged among the most important motivating factors in both groups. This is consistent with the findings of Clancy et al. (8) and Kinczel et al. (9), who emphasize that intrinsic and extrinsic motivational factors jointly shape the relationship to physical activity. An interesting observation is that sports major students attributed greater importance to external reinforcement sources — including professional guidance (d = 1.11, large effect), entertainment (d = 0.71), parental opinion (d = 0.65), and peer networks (d = 0.55) — compared to non-sports majors. This difference may reflect the social norms of a sports science programme, where peer and professional networks naturally play a more prominent role, though this interpretation goes beyond what the present data can confirm.

H4 was confirmed. Parental sports background was not a significant direct predictor of group membership, with negligible to small and non-significant effect sizes for both father (d = 0.10) and mother (d = 0.27) variables. This partially contrasts with the modeling role emphasized by Fintor and Szabó (13 and Herpainé Lakó (15) in earlier developmental stages, and is more consistent with Fenyves et al. (16), who found that family played a diminishing motivational role among Hungarian university students. The binary operationalization of parental sports background, however, in the present study may not fully capture the complexity of family socialization processes. More nuanced instruments would be needed to draw stronger conclusions about the role of parental modeling at this life stage. Nevertheless, secondary associations between a sporting father model and reliance on parental and professional opinions (*p* = 0.039 and *p* = 0.027 respectively) suggest that early family socialization may leave an indirect imprint on how receptive students are to social influences in sport later in life.

The similar BMI values across both groups (d = −0.00, negligible) represent a finding that requires careful interpretation. Despite substantially lower cardiovascular fitness, non-sports major students maintained comparable body mass indices to their sports major peers. Rather than interpreting this as direct evidence of health-conscious behavior among non-sports majors, a more cautious reading would note that BMI is a limited health indicator that does not distinguish between fat mass and muscle mass, and that weight maintenance may reflect a variety of factors — dietary habits, metabolism, or socioeconomic conditions — rather than intentional health behavior. This limitation of BMI as a health proxy is acknowledged and should be considered when interpreting this finding.

Non-sports major students demonstrated a large cardiovascular fitness deficit relative to their sports major peers, even after controlling for age, sex, and BMI. At the same time, their subjective health image appeared disconnected from functional movement quality, suggesting a health literacy gap. These observations may serve as starting points for future research on health promotion strategies targeting non-sports major students in higher education; however, this study's cross-sectional design does not allow effectiveness conclusions.

### 4.1 Limitations of the study

Several limitations must be acknowledged. First, the cross-sectional design restricts the ability to draw causal conclusions or to track how physical activity patterns evolve over time. Second, the sample size is relatively small (*N* = 146) and unequally distributed across groups (*n* = 50 sports major vs. *n* = 96 non-sports major), which limits the statistical power of certain comparisons and the generalizability of the findings to broader student populations. Third, the internal consistency of the questionnaire was only marginally acceptable (Cronbach's alpha = 0.658), falling below the commonly recommended threshold of 0.70 (18), which represents a methodological limitation that should be addressed in future studies through instrument development and validation. Fourth, BMI is a widely recognized but limited proxy for health status, as it does not differentiate between fat mass and lean body mass, and may therefore misrepresent the true body composition of physically active individuals. Fifth, self-reported questionnaire data are inherently susceptible to response biases, including social desirability effects, whereby participants may report more favorable health behaviors or self-perceptions than are actually present. Accordingly, the findings apply specifically to recreationally active non-sports major students and cannot be generalized to fully sedentary university populations.

## 5 Conclusions

This study examined the physical status, subjective self-evaluation, and sports motivation of sports major and non-sports major university students at a Hungarian higher education institution, adding to research on the behavioral and physical determinants of active living during the transition into early adulthood. Sports major students demonstrated substantially higher cardiovascular fitness than their non-sports major peers, as reflected by significantly greater estimated VO2max values (d = 1.26, large effect). Sports major students also reported more favorable subjective self-evaluations across all examined dimensions, with effect sizes ranging from small to large. The correlation analyses indicated that subjective self-evaluations were only partially grounded in objective physical indicators: cardiovascular endurance and body composition showed moderate associations with self-reported fitness and health, whereas functional movement quality, as measured by the FMS, showed no significant correlation with any subjective variable and did not appear to be incorporated into students’ subjective perception of health. BMI values were nearly identical across groups; this finding should be interpreted cautiously, as weight maintenance may reflect various factors — including dietary habits, metabolism, or socioeconomic conditions — rather than intentional health-conscious behavior. Parental sports background was not a direct determinant of group membership, although secondary associations suggest that early family socialization may indirectly shape students’ receptivity to social influences in sport. These findings should be interpreted within the constraints of the study's design. The cross-sectional design precludes causal inference, the marginal internal consistency of the questionnaire (Cronbach's alpha = 0.658) represents a methodological limitation, and the relatively small and unequally distributed sample (*N* = 146) limits generalizability. Within these limitations, the study contributes two observations relevant to future research. First, the absence of functional movement quality from students’ subjective health image points to a potential gap in health literacy that warrants further investigation. Second, the comparable BMI values across groups — despite large differences in cardiovascular fitness — represent an interesting pattern; however, given the limitations of BMI as a health proxy and the cross-sectional design, this observation may tentatively point toward some degree of weight-related health awareness among non-sports major students — a potentially meaningful question for future physical education and health promotion research. Longitudinal studies with larger, more representative samples and a psychometrically validated self-report questionnaire instrument are needed to examine these associations with greater precision.

## Data Availability

The raw data supporting the conclusions of this article will be made available by the authors, without undue reservation.
